# Mental imagery in adolescent PTSD patients after child abuse: a comparison with matched healthy controls

**DOI:** 10.1186/s12888-022-03706-8

**Published:** 2022-01-27

**Authors:** Regina Steil, Anne Fischer, Jana Gutermann, Rita Rosner

**Affiliations:** 1 Department of Clinical Psychology and Psychotherapy, Institute of Psychology, Goethe University Frankfurt, Varrentrappstr. 40-42, 60486 Frankfurt am Main, Germany; 2 Department of Psychology, Catholic University Eichstätt-Ingolstadt, Ostenstr. 25, 85072 Eichstätt, Germany

**Keywords:** PTSD, Negative mental images, Positive mental images, Childhood abuse, Adolescence, Childhood

## Abstract

**Background:**

Intrusive mental imagery (MI) plays a crucial role in the maintenance of posttraumatic stress disorder (PTSD) in adults. Evidence on the characteristics of MI in adolescents suffering from PTSD is sparse. The aim of this study was to thoroughly assess MI in an adolescent sample suffering from PTSD after the experience of childhood sexual abuse and/or childhood physical abuse (CA).

**Methods:**

Thirty-two adolescents with a primary diagnosis of PTSD after CA and 32 adolescents without any mental disorder and without a history of CA, matched for age and gender, completed questionnaires assessing the characteristics of negative and positive MI, as well as images of injury and death that lead to positive emotions (ID-images).

**Results:**

The PTSD group reported significantly more frequent, more vivid, more distressing and more strongly autobiographically linked negative MI compared to the control group. Although positive MI was highly present in both groups (PTSD: 65.6%; controls: 71.9%), no significant differences emerged between the two groups regarding the distinct characteristics of positive MI. The frequency of the ID-images did not significantly differ between the two groups (PTSD: 21.9%; controls: 9.4%), although the ID-images were more vivid in the PTSD group.

**Discussion:**

Negative MI appears to be crucial in adolescent PTSD, whilst positive MI are unexpectedly common in both the PTSD and the control group. The role of positive MI as well as that of ID-images remain unclear. Specific interventions for changing negative MI that are tailored to the developmental challenges in adolescents with PTSD should be developed.

**Trial registration:**

Some of the PTSD patients in this study were also part of a randomized controlled trial on Developmentally adapted Cognitive Processing Therapy (D-CPT). This trial was registered at the German Clinical Trial Registry (GCTR), DRKS00004787, 18 March 2013.

**Supplementary Information:**

The online version contains supplementary material available at 10.1186/s12888-022-03706-8.

## Background

Mental imagery (MI) refers to image-based thoughts occurring in multiple sensory modalities in contrast to verbal-based thoughts [1]. Thereby, mental images are defined by “representations and the accompanying experience of sensory information without a direct external stimulus” ([2], p.560) and are often described as “seeing with the mind’s eye” or “hearing with the mind’s ear” [3, 4].

In adults, unpleasant or negative MI is a frequent phenomenon which exerts a substantial negative impact on well-being in various mental disorders such as posttraumatic stress disorder (PTSD) or depression; this has been confirmed in a large body of studies (for a review see [1, 5–7]). Patients with mental disorders often experience negative MI as vivid, uncontrollable and highly distressing episodes [7], and, as a result, higher levels of vividness are associated with greater levels of distress [8–10]. Negative MI often refers to intrusive memories of a negative autobiographic event [11], however, it may also be linked to non-memory-based scenes or even future-oriented feared disasters such as so-called involuntary time travel [4]. In PTSD, intrusive images are seen as being generated at points of maximum emotional disturbance during a traumatic event, leading to impeded memory encoding [12]. However, despite intrusive imageries that represent fragments of the trauma, there are also images that do not represent fragments of any traumatic experience [13].

MI can be experienced either from an external perspective that involves the observation of the self as through the eyes of others, or the field-perspective (“trough my own eyes”, [5]). In adult PTSD, experiencing intrusive images with a field-perspective appears to be related to more intense emotions, dissociation, and physiological sensations, as well as altered psychological states similar to those experienced during the traumatic event [7, 14]. In contrast, the observer-perspective is presumed to be related more to details concerning spatial relations or the periphery (for a review see [7]). Adult PTSD patients tend to use the observer- more frequently than the field-perspective [14] which can be seen as an avoidance mechanism to escape burdening emotions [15].

A distinct phenomenon in the realm of MI is termed “flash-forwards”; this comprises images regarding prospective self-harm or suicidal ideation that are produced intentionally and, in turn may, aggravate suicidal ideation in adult patients suffering from depression or borderline personality disorder [16–18]. The important role of prospective imagery is underlined by findings that imagining a future scenario causally increases the likelihood that the person will, in turn, act these out as imagined [19, 20].

Recent studies have described a different aspect of unpleasant images of injury and death (“ID-images”) in the realms of adult depression or borderline personality disorder [4, 16, 21]. According to the definition by Weßlau et al. [4], these ID images refer to images with objectively negative content (e.g., the own suicide or the death of another person), but go along with rather positive feelings such as comfort or relief instead of distress during their experience. Thereby, the number of ID-images increases with the increasing level of depression severity. Furthermore, ID-images have been described as being pleasant and controllable, so one may speculate that they are produced intentionally as a cognitive escape strategy [4]. However, to date, little is known about ID-images or flash-forwards in mental disorders beyond depression or borderline personality disorders, as well as the role ID-images play in adolescent samples.

In contrast, positive or pleasant mental images that may be linked to positive autobiographic experiences occur frequently in non-mentally ill samples [22]. Contrary to this, individuals suffering from a mental disorder report less frequent or less vivid positive MI in comparison to healthy controls [23–25]. However, studies examining interventions that are intended to train positive prospective MI such as positive cognitive bias modification (CBM), show that depressive (e.g., [26]) as well as PTSD symptoms (e.g., [27]) in adults can be reduced and that the general mental health might be improved (for an overview see [23]). Furthermore, other imagery-based interventions such as imagery rescripting in cognitive behavioral treatments have been shown to be highly effective in reducing symptom severity in adults suffering from different mental disorders such as PTSD or social anxiety disorder (e.g. [28, 29]).

However, in contrast to the outlined adult literature, empirical evidence regarding MI in the realms of child and adolescent psychopathology is rather sparse (for a review see [12, 13]) or non-existent with regard to ID-images. This is surprising since many mental disorders in adults can be traced back to childhood or adolescence, whilst image-based processing regarding various cognitive, procedural or epistemic domains is even more prominent in children and adolescents than in adults [30]. In adult PTSD, the nature of intrusive MI is better understood [7, 11, 31], however, in adolescence little is known regarding the realm of mental images linked to PTSD. Few studies have investigated the role of negative MI in traumatized children and adolescents; these studies are summarized below.

Evidence regarding the role of children’s intrusive imagery stems from Holmes et al. [32] who examined the content of intrusive imagery, as well as its relation to the development and persistence of posttraumatic stress symptoms, in *N* = 76, 10 to 11 year-old school children who witnessed the terrorist attacks of September 11th 2001 on television. About one third of the children reported experiencing intrusive images 2 months post the terrorist attacks while 25% were experiencing these after 6 months. Furthermore, they found that negative MI, in conjunction with the peri-traumatic experience of threat, predicted short-term and long-term posttraumatic stress symptoms. Thus, imagery seems to predict traumatic responses in children but only if the child had been extremely frightened and feared a threat of life during the traumatic event. However, the small age range and the analog nature of the sample limit the general implications of this study.

Eksi et al. [33] investigated the sensory characteristics of vivid intrusive memories and mental images in a case series of five children suffering from PTSD after an earthquake in Turkey. They showed that negative intrusive images contain different sensory qualities in child PTSD: three children reported visual negative mental images, one child experienced auditory images while one child reported an olfactory experience. McKinnon et al. [34] examined the relationship between intrusive memories and peri-traumatic processing strategies (e.g., the data-driven processing of perceptual/physical aspects of the trauma) in *N* = 75 children aged 7 to 16 years. They demonstrated that peri-traumatic encoding strategies impede the processing of traumatic memories which is in line with the cognitive models of PTSD [12].

Meiser-Stedman et al. [35] focused on investigating the mechanisms associated with the development of intrusive memories after experiencing a life-event in an adolescent community sample. Firstly, they showed that intrusive memories are a common phenomenon in adolescents and not specific to experiencing a traumatic event. In line with McKinnon et al. [34], the quality of the memory of the event (e.g., sensory) seems to mediate, partially, the relationship between the peri-event affect (e.g., sad, angry, scared, ashamed, helpless) and the intrusive memories. Interestingly, it was not only fear that was found to be related to the frequency of intrusive memories, but also other emotions such as anger or sadness. However, the latter study focused on MI after a life-event and not specifically a traumatic event or PTSD. Evidence for adolescents with PTSD in a clinical context is lacking, so that being able to draw valid conclusions about negative MI in adolescents suffering from PTSD is definitely called for.

To the best of our knowledge, no study has so far examined the role of MI in adolescents suffering from PTSD after childhood sexual abuse and/or childhood physical abuse (CA) as compared with a control group (CG) without mental disorders and without an abuse history, matched for age and gender. This is also the first study to investigate the nature of positive and negative MI as well as ID-images, in an adolescent sample suffering from PTSD after CA, stating the following hypotheses:

### Negative MI

1. We expect the PTSD group to experience negative MI significantly more frequently, more vividly, more uncontrollably, as well as more distressingly than the the CG.
2. We hypothesize that, relative to the CG without mental disorders, adolescent participants suffering from PTSD would a) experience concomitant emotional states, such as anxiety or guilt related to the unpleasant image significantly more intensely, b) experience a greater autobiographical association of the image and c) report a significantly worse mood in response to negative MI.
3. In exploratory analyses, we analyze which perspective adolescents adopt in their images and how often different behavioral strategies (e.g., I drink alcohol; I hurt myself by cutting; I have eating binges) in response to negative MI were chosen by adolescents suffering from PTSD in comparison to the CG.

### Positive MI

(4) We hypothesize that adolescents suffering from PTSD would experience positive MI significantly less frequently, as well as less vividly, than the CG.
(5) Furthermore, we examine a) how closely the images are related to an autobiographic event and b) whether there are mood changes in response to the positive MI by comparing the two groups.

### ID-images

(6) Concerning the ID-images we wish to determine whether adolescents suffering from PTSD experience these images and, additionally, whether these ID-images are significantly more frequent and more vivid than the ID-images that might be experienced by healthy adolescents.
(7) By means of exploratory analyses, we examine whether ID-images lead to improved mood in adolescent patients with PTSD, in line with the findings by Weßlau et al. [4].

## Methods

### Participants

#### PTSD group

The PTSD group consisted of 32 adolescents with a primary diagnosis of CA-related PTSD according to the Diagnostic and Statistical Manual of Mental Disorders (DSM-IV-TR, [36]) with a lowered threshold for avoidance symptoms (a minimum of one re-experiencing criteria, two avoidance symptoms and two hyperarousal symptoms were sufficient for PTSD diagnosis [37]). In line with the American Psychological Association [38], the definition of child sexual abuse comprised the sexual assault of a minor or sexual activity between a minor and an older person with the older person using the dominant position to coerce or force the younger person. The definition of child physical abuse included the intentional injury of a child by a parent or caretaker, e.g., by striking, kicking, beating, biting or any action that leads to physical injury [38].

The patients had been referred by child-welfare agencies, social workers, school counselors, caregivers, local child and adolescent psychiatrists or psychotherapists for treatment in either a pilot treatment study [39] or a multicenter randomized controlled trial (RCT) [40], comparing a developmentally adapted treatment of Cognitive Processing Therapy (D-CPT) to a wait-list condition with treatment advice (WL/TA). The PTSD-diagnosis was established by trained and clinically experienced raters, using the Clinician Administered PTSD Scale - Child and Adolescent version in German (CAPS-CA, [41]; in German IBS-KJ [42]). Comorbid disorders as well as the absence of excluded disorders were established using the semi-structured Structured Clinical Interview for DSM-IV Axis I (SKID I [43]; in German [44]) and the Borderline section from the Structured Clinical Interview for DSM-IV Axis II (SKID II [45]; in German [46]) as well as relevant sections (e.g., separation anxiety disorder, conduct disorders, tic disorders, mutism, enuresis, and encopresis) from the Diagnostic Interview for Mental Disorders in Childhood and Adolescence (Kinder-DIPS [47]). Further inclusion criteria comprised being between 14 and 21 years old, having sufficient knowledge of the German language as well as no ongoing abuse. Exclusion criteria comprised a lifetime diagnosis of a psychotic disorder, pervasive developmental disorder, substance-related or organic mental disorders, bipolar disorder, suicidal ideation and simultaneous psychological or pharmacological treatment.

The mean age of the patients was *M* = 17.6 years (*SD* = 1.93, range: 14–21, 84.4% women). A total of 8 patients (25%) had one comorbid axis I disorder, 8 patients (25%) suffered from two comorbid axis I disorders, and 11 patients had a diagnosis of 3 comorbid disorders (34.4%), while 5 patients (15.6%) suffered from 4 to 6 comorbid disorders. The abuse characteristics, as well as the symptom severity of the sample, are shown in Table 1.

**Table 1:** Abuse characteristic and symptom severity of the PTSD group (*n* = 32)

Abuse or PTSD characteristics	Results
**History of sexual abuse,** ***N (%)***	28 (87.5)
Number of sexual abuse clusters, *M (SD)*	2.19 (2.28)
Age in years at the start of the sexual abuse, *M (SD)*	11.43 (3.85)
Duration in months of the sexual abuse, *M (SD)*	25.3 (30.26)
**History of physical abuse,** ***N (%)***	23 (71.9)
Number of physical abuse clusters, *M (SD)*	2.69 (3.06)
Age in years at the start of the physical abuse, *M (SD)*	8.87 (5.60)
Duration in months of the physical abuse *M (SD)*	68.91 (54.80)
**CAPS-CA sum score,** ***M (SD)***	68.78 (29.44)
Re-experiencing, *M (SD)*	20.9 (9.56)
Avoidance, *M (SD)*	26.63 (12.51)
Hyperarousal, *M (SD)*	21.28 (12.07)

Note: *CAPS-CA* Clinician-Administered, *PTSD* Scale for Children and Adolescents

Additionally, we conducted exploratory analyses to test for correlations between the psychopathologies in the PTSD group and MI characteristics. The results can be retrieved from Additional File 1.

### CG without mental disorders and without a history of CA

A total of 32 adolescents and young adults without any mental disorder and matched for age and gender to the PTSD group were recruited by means of local advertisements and notice boards. The absence of any axis I disorders was reassured by conducting the SCID I [43, 44] and the Kinder-DIPS [47] interviews prior to study intake which were conducted by raters holding a Bachelor degree in psychology. Furthermore, a history of CA was an exclusion criterion as well as an insufficient knowledge of the German language for recruitment to the CG.

### Procedures

The study was approved by the local ethics committee of the Department of Medicine. Prior to any assessment, participants and their parents (for participants aged below 18 years) provided written informed consent. After the semi-structured interview for the establishment of diagnoses, the participants completed the questionnaires. Participants from the CG received 15€ for their participation.

### Measures

#### Questionnaire for mental images in adolescents

For this study, a questionnaire assessing retrospective positive and negative MI, as well as future-oriented ones, was developed on the basis of the questionnaires used in prior studies by Schreiber & Steil [48] and Weßlau et al. [4]. These, in turn, were based on the semi-structured interview used by Hackmann et al. [49]. The questionnaire begins with an introduction defining what is meant with mental images to ensure that the participants understand the subsequent sections. This introduction is followed by three separate parts: positive MI (named pleasant images), negative MI (named unpleasant images) and ID-images (named images of injury and death with positive feelings). In each of these three sections, the individual is first asked whether or not he, or she, experiences mental images relating to the particular category. In the event of a positive response, the individual is asked to describe an image of the category in his/her own words that is most frequent or most distressing to them. In the sections for negative MI and the section for ID-images, the description is followed by further ratings of “yes” or “no” regarding the *content* of the described image, for instance “in this image, I experience physical violence from another person (negative image)” or “in this image, I try to commit suicide (ID-image)”. Subsequently, in all three sections, referring to the earlier described mental image, participants are asked to indicate the *frequency* of occurrence as well as the *vividness* of the image in the previous 2 weeks on a 0 to 100 visual analog scale. An item then assesses whether the image refers to the past, the present or the future; participants can also choose “I don’t know”. The next item asks about the *change of mood* in response to the mental image on an analog scale ranging from worse to better with 0 as an anchor for no mood alteration. Lastly, the adolescents are asked whether the positive or negative image is related to an *autobiographic event* and indicate their response on an analogue scale ranging from 0 (not at all) to 100 (totally). In the event of an affirmative answer, the adolescents are asked for their age at the time, when the autobiographic event took place, but this was only applied in the section concerning negative MI.

The section examining negative MI is more detailed than the section concerning positive MI or ID-images and, therefore, entails more questions. Additionally, the adolescents are asked to rate 13 possibly *concomitant emotions* on a 0 to 100 visual analog scale (e.g., “In the image, I feel ashamed”/ “I feel disgust”). Moreover, participants indicate how *distressing* the negative image is on a visual analog scale from 0 (not at all) to 100 (extremely), as well as how *uncontrollable* the image is from 0 (uncontrollable) to 100 (controllable). Subsequently, the adolescents are asked for their *strategies* in response to the unpleasant image. They indicate the extent of use of 12 common strategies (such as “In response to the image, I drink alcohol/do drugs”) on a bipolar visual analog scale ranging from 0 (never) to 100 (always). The next item assesses the individual’s *perspective* within this image; they indicate whether they see themselves “through their own eyes” or rather take an observer-perspective (“through the eyes of other people”).

### Clinician-administered PTSD scale for children and adolescents (CAPS-CA)

The CAPS-CA [41, 42] is a widely-used structured clinical interview for the assessment of PTSD according to the DSM-IV [36] and its severity; this was used to establish the PTSD diagnosis for the study intake (PTSD group only). In the present sample, the internal consistency of the CAPS-CA was excellent with *α* = .960.

### University of California Los Angeles PTSD Reaction Index for DSM-IV (revision 1; UCLA-PTSD-RI)

The German version [50] of the UCLA-PTSD-RI [51] was used in this study to assess the self-reported trauma exposure as well as the PTSD symptoms and yields good psychometric properties (e.g., [52]). The questionnaire consists of two parts: following a brief lifetime trauma screen, the A1 and A2 criteria of DSM-IV PTSD are examined, followed by 22 items assessing the frequency of PTSD symptoms during the past month (rated from 0 = *not at all* to 4 = *most of the time*). In our sample, the questionnaire had an excellent reliability with *⍺* = .954.

### Data analysis

Missing values were screened and analyzed using Little’s MCAR-Test. For the sections regarding negative MI (1.5%, χ2(152) = 157.01, *p* = .37), positive MI (0.6%, χ2(3) = 0.98, *p* = .81) as well as ID-images (3.8%, χ2(3) = 3.49, *p* = .32), the percentage of missing values for all adolescents reporting experiencing the respective images were lower than 5% and were proved to miss completely at random. For the UCLA-PTSD-RI (0.01%; χ2(21) = 32.38, *p* = .05) data were also missing completely at random. If data were missing completely at random, they were replaced by using the EM algorithm.

By using a χ^2^-test, the two groups were compared regarding demographic variables. However, due to the lack of variance homogeneity, we conducted a Welch’s t-test to compare the mean scores on the UCLA-PTSD-RI scales [53]. For the main analyses, only the data for adolescents who had indicated experiencing negative MI, positive MI or ID-images were analyzed, resulting in different and, partly, small sample sizes regarding the analyses for the respective image category. In order to analyze the hypotheses, multivariate analysis of variance (MANOVAs) as well as χ^2^-tests were conducted. In cases of violated assumptions (e.g., sample sizes below 5), the Fisher-Yates-test was computed instead of the χ^2^-tests. Due to inevitably unequal sample sizes for some of the analyses, the non-parametric Mann-Whitney-U-test, instead of the MANOVA or independent t-tests, was computed, as this test is classified as being robust to unequal sample sizes [54]. The effect size estimate for the Mann-Whitney-U-test is computed as *r* [55]*.* With regard to multiple comparisons (e.g. concerning the emotional states in response to negative MI), analyses were adjusted by using the Bonferroni-correction. All statistical analyses were computed with IBM SPSS Statistics 27 for Windows.

## Results

### Descriptive data on psychopathology

Table 2 depicts the demographic data as well as the results of group comparisons regarding the UCLA. Since the participants were matched for age and gender, they were only compared regarding educational level and immigration background. A χ^2^-test revealed that the two groups differed significantly regarding their educational level, including significantly more participants with a high-school degree in the CG when compared to the PTSD group. Furthermore, a significant difference regarding the immigration background emerged, yielding more participants with an immigration background in the CG in comparison to the PTSD group. Adolescents suffering from PTSD yielded significantly higher scores on the UCLA-PTSD-RI in comparison to adolescents without a mental disorder.

**Table 2:** Demographic characteristic and clinical measures

	PTSD (*n* = 32) n (%) or Mean (*SD*)	CG (*n* = 32) n (%) or Mean (*SD*)	Statistics
Educational level			χ^2^(_1,64_) = 15.52*
Still going to school	16(50.0%)	12 (37.5)	
No degree	2 (6.3%)	0 (0.0%)	
Basic degree	6 (18.8%)	2 (6.3%)	
Advanced degree	6 (18.8%)	3 (9.4%)	
High-school degree	2 (6.3%)	15 (8.5%)	
Immigration background	7 (21.9%)	16 (50.0%)	χ^2^(_1,64_) = 5.50*
UCLA-PTSD-RI			
Sum score_a_	41.38 (12.11)	8.41 (6.55)	*t*(49.18) = 13.27; *d* = 9.97***
*Re-experiencing*_a_	13.16 (4.86)	1.48 (2.44)	*t*(47.26) = 11.93; *d* = 3.95***
*Avoidance*_a_	15.50 (6.23)	2.89 (3.14)	*t*(47.37) = 10.04; *d* = 5.06***
*Hyperarousal*_a_	12.72 (4.01)	4.04 (3.14)	*t*(56.73) = 9.32; *d* = 3.64***

Note: *PTSD* posttraumatic stress disorder; *CG* control group without mental disorders; *UCLA-PTSD-RI* University of California Los Angeles PTSD Reaction Index for DSM-IV; _a_  *n* = 27 in the CG and  *n* = 32 in the PTSD group; ^*^p < .05, ^***^p < .001

### Negative MI

Overall, 29 (90.6%) patients suffering from PTSD reported experiencing negative MI, whereas this was reported in only 8 (25%) adolescents from the CG, thus, revealing a significant difference between the groups (χ^2^(1,64) = 28.25, *p* < .001). Table 3 shows the categories regarding the content of the negative MI. The content of the negative MI in the PTSD group is highly trauma-related (e.g., experiencing physical violence (82.8%), sexual violence (75.9%) and psychological abuse (55.2%)). Due to the small sample size for several content categories in the CG, as well as missing values in the PTSD group, further analyses concerning the content were not feasible.

**Table 3:** Content of negative mental images according to default categories

	PTSD (*n* = 29)	CG (*n* = 8)
Content In this image …	*n (%)*	*n (%)*
… I will be left by an important person	6 (20.7)^a^	2 (25)
… I look like a loser	5 (17.2)^a^	1 (12.5)
… another person dies	4 (13.8)^a^	2 (25)
… I die	8 (27.6)^b^	0
… I experience physical violence by others	24 (82.8)	1 (12.5)
… I experience psychological violence by others (e.g., mobbing)	16 (55.2)^d^	0
… I experience sexual violence	22 (75.9)	0
… I hurt myself	5 (17.2)	0
… I kill myself	3 (10.3)	0
… get killed	6 (20.7)	0
… I am in an accident	2 (6.9)	1 (12.5)
… someone else is in an accident	3 (10.3)^b^	2 (25)
… I get persecuted	11 (37.9)^b^	0
… I see a person who triggers negative feelings in me	23 (79.3)^e^	1 (12.5)
… I see a place in which I feel uncomfortable	23 (79.3)	3 (37.5)

Note: *PTSD* posttraumatic stress disorder; *CG* control group without mental disorders; ^a^*n* = 27, ^b^*n* = 28, ^c^*n* = 25, ^d^*n* = 25, ^e^*n =* 26 in the PTSD group

#### Frequency, vividness, level of distress and controllability regarding negative MI (hypothesis 1)

Bonferroni-corrected (*p* = .012) Mann-Whitney-U-tests revealed that the PTSD group reported to have experienced a significantly higher *frequency* of negative MI in previous 2 weeks compared to the CG (*Mdn* = 9, *Mdn* = 1; *U* = 18.5, *z* = − 3.62, *p* < .001, *r* = −.60). Furthermore, adolescents with PTSD reported significantly higher *levels of distress* caused by negative MI (*Mdn* = 90, *Mdn* = 28; *U* = 24.0, *z* = − 3.51, *p* < .001, *r* = −.58) as well as a greater *vividness* of negative MI (*Mdn* = 80, *Mdn* = 40; *U* = 42.5, *z* = − 2.74, *p* = .005 *r* = −.45) relative to adolescents without a mental disorder. No significant group difference emerged regarding the extent of controllability of the negative MI (*Mdn* = 25, *Mdn* = 30; *U* = 92.0, *z* = − 0.89, *p* = .19, *r* = −.15).

#### Concomitant emotional states in response to negative MI, autobiographical association and mood change (hypothesis 2)

The Mann-Whitney-U-Test was used for the investigation of emotional responses to the negative image. Seven of the 13 emotional states yielded significant differences concerning their intensity between the PTSD group and the CG after Bonferroni correction. Table 4 depicts the medians and the test statistics for each emotional response. Overall, adolescents suffering from PTSD reported significantly greater feelings of *self-hate, disgust, helplessness*, *guilt*, *worthlessness, anxiety, anger* and the *feeling of being contaminated*. No differences between the groups emerged for *shame, sadness, loneliness, jealousy* or *numbness.*

**Table 4:** Concomitant emotional states in adolescents experiencing negative images

	PTSD (*n* = 29) Median	CG (*n* = 8) Median	Statistic
Concomitant emotion			
shame	70	5	*U* = 53.5, *z* = − 2.33; *p* = .009, *r* = −.38
self-hate	70	0	*U* = 43.5, *z* = − 2.73; *p* = .002, *r* = −.45*
disgust	90	0	*U* = 17.5, *z* = − 3.76; *p* < .001, *r* = −.62*
helpless	100	80	*U* = 29.0, *z* = − 3.56; *p* < .001, *r* = −.59*
guilty	70	0	*U* = 38.5, *z* = − 2.91; *p* = .001, *r* = −.48*
worthless	90	0	*U* = 20.0, *z* = − 3.73; *p* < .001, *r* = −.61*
anxious	100	37.5	*U* = 27.5, *z* = − 3.41; *p* < .001, *r* = −.56*
sad	100	70	*U* = 56.0, *z* = − 2.38; *p* = .01, *r* = −.39
angry	80	2.5	*U* = 39.0, *z* = − 2.89; *p* = .001, *r* = −.48*
lonely	90	37.5	*U* = 45.5, *z* = − 2.67; *p* = .040, *r* = −.44
jealous	0	0	*U* = 72.0, *z* = − 2.01; *p* = .040, *r* = −.33
feeling of being contaminated	90	0	*U* = 36.5, *z* = − 3.06; *p* = .001, *r* = −.50*
numb	80	10	*U* = 67, *z* = − 1.85; *p* = .033, *r* = −.30

Note: *PTSD* posttraumatic stress disorder; *CG* control group without mental disorders; *significant after Bonferroni-correction: adjusted *p = .*05/13 = .004

Negative MI was found to be significantly more strongly related to an autobiographical event in the PTSD group than the CG (*Mdn =* 100, *Mdn* = 30*; U* = 61.5, *z* = − 2.36, *p* = .012, *r* = −.43). In contrast, even though the PTSD group reported their mood to be worse following a negative image (*M* = − 27.43, *SD* = 25.35, vs. *M* = 1.22, *SD* = 0.42 in the CG), the Mann-Whitney-U-test did not find a significant difference in comparison to the CG concerning mood alteration after negative MI (*Mdn =* − 40.0, *Mdn = −* 10.0*; U* = 78.5, *z* = − 1.41, *p* = .082, *r* = −.23).

#### Perspective and behavioral responses to the negative MI (hypothesis 3)

On an exploratory level, we analyzed which perspective adolescents adopt in their images and how often different behavioral strategies are applied in response to a negative MI. For the imagery perspective, 7 (25.0%) PTSD patients reported experiencing the negative image from the observer-perspective (vs. *n* = 1 12.5% in the CG), while 16 (57.1%) adolescents suffering from PTSD stated experiencing it from their own perspective (vs. *n* = 7, 87.5% in the CG). The resulting 5 patients indicated experiencing negative images from both perspectives, while no participant in the CG reported having both perspectives. Since 3 (50.0%) cells had an expected frequency below 5, statistical comparisons between the two groups on this variable using χ^2^-test were not feasible.

Table 5 depicts the medians for rating the elicited behavioral responses to the negative MI. The distributions differed between both groups in 6 out of 12 items, Kolmogorov-Smirnov *p* < .05. The Bonferroni-corrected Mann-Whitney-U tests showed that traumatized adolescents scored significantly higher only for “I have a fit of rage” compared to adolescents without a mental disorder. As indicated in Table 5, none of the other comparisons revealed significant differences between the groups.

**Table 5:** Behavioral responses and group differences in medians between PTSD and CG

Behavioral response	PTSD (*n* = 29) *Mdn*	CG (*n* = 8) *Mdn*	Statistic
I detract myself	80.0	77.5	*U* = 96.5, *z* = −.73, *p* = .24, *r* = −.12
I try very hard to control my emotions	80.0	60.0	*U* = 63.5, *z* = − 1.92, *p* = .03, *r* = −.32
I do something good to myself	50.0	47.5	*U* = 116.0, *z* = .00, *p* = .50, *r* = .00
I drink alcohol/do drugs	0	0	*U* = 94.5, *z* = − 1.05, *p* = .15, *r* = −.17
I ruminate about the image	50.0	60.0	*U* = 97.5, *z* = −.69, *p* = .25, *r* = −.11
I talk to somebody	10.0	55.0	*U* = 77.0, *z* = − 1.51, *p* = .07, *r* = −.25
I hurt myself (e.g., through cutting myself)	0	0	*U* = 96.0, *z* = − 1.24, *p* = .27, *r* = −.20
I punish myself (e.g., I forbid myself to do something that I was looking forward to)	0	0	*U* = 80.0, *z* = − 1.77, *p* = .08, *r* = −.29
I think of committing suicide	0	0	*U* = 72.5, *z* = − 1.84, *p* = .03, *r* = −.39
I have eating or vomiting attacks	0	0	*U* = 89.0, *z* = − 1.23, *p* = .13, *r* = −.20
I endanger myself (e.g., hazardous driving, unprotected sex)	0	0	*U* = 104.0, *z* = −.94, *p* = .47, *r* = −.15
I have a rage attack	20.0	0	*U* = 44.0, *z* = − 2.86, *p* = .02, *r* = .47*

Note: *PTSD* posttraumatic stress disorder; *CG* control group without mental disorders; *significant after Bonferroni-correction: adjusted *p = .*05/12 = .004

### Positive MI

Regarding the experience of positive mental images, there was no significant difference between the two groups (χ^2^(1,64) = .29, *p* = 59); a total of 21 (65.6%) adolescents with PTSD responded affirmatively to the presence of positive images while 23 (71.9%) participants of the CG reported experiencing positive images. When comparing the two groups regarding the *frequency* (*M*_*PTSD*_ = 10.43, *SD =* 11.80; *M*_*CG*_ = 5.76, *SD =* 6.47), *level of vividness* (*M*_*PTSD*_ = 73.57, *SD =* 26.70; *M*_*CG*_ = 65.43, *SD =* 27.38) as well as *associated autobiographical event* (*M*_*PTSD*_ = 63.57, *SD =* 40.90; *M*_*CG*_ = 79.78, *SD =* 30.39)*,* a MANOVA revealed no significant main effect of group (*F*(3,40) = 1.90; *p* = .15; partial η^2^ = .13, Wilks λ = .88). Furthermore, we found no differences between the two groups regarding an altered mood following the positive image (*M*_*PTSD*_ = 30.00, *SD* = 26.27; *M*_*CG*_ = 32.83, *SD* = 11.59, *t*(26.94) = −.45, *p* = .65).

### ID-images

Regarding the ID-images, 7 (21.9%) adolescents from the PTSD group and 3 (9.4%) adolescents from the CG reported experiencing ID- images, while this difference was not found to be significant using the Fishers-Yates-test (*p* = .18). Further analyses of the adolescents responding affirmatively to the item of the presence of ID-images revealed that the PTSD-group did not experience ID-images significantly more frequently than the CG (*Mdn*_*PTSD*_ *= 2, Mdn*_CG_ = 1; *U* = 7.00, *z* = −.82; *p* = .41, *r* = −.26). However, the ID-images were significantly more vivid in adolescents suffering from PTSD compared to the CG (*Mdn*_*PTSD*_ = 80, *Mdn*_CG_ = 10; *U* = .50, *z* = − 2.29; *p* = .02, *r* = −.73). No significant difference between the groups was detected for the alteration of mood following the ID-image (*Mdn*_*PTSD*_ = − 4.44, *Mdn*_CG_ = − 20; *U* = 6.5, *z* = −.92; *p* = .36, *r* = −.29). A closer inspection regarding the described content of the ID-images showed that 3 adolescents from the PTSD group reported that the ID-image consists of self-injurious behavior (intentional self-harm *(n* = 1), an accident leading to breaking all my bones (n = 1) or suicide *(n* = 1)). The participant from the PTSD group who reported ID-images related to self-harm felt worse afterwards, the one with ID-images of his/her suicide experienced no mood change while the participant who reported the accident-image experienced a better mood. One adolescent reported experiencing three different categories of ID-images, namely intentional self-harm, committing suicide and beating up her ex-boyfriend, followed by a slight amelioration of mood. Two adolescents reported ID-images consisting of hurting, raping or killing the perpetrator that were associated with feelings of revenge. Interestingly, one of the latter reported a better mood and the other one a worse mood in reaction to their ID-image. The last PTSD patient had images of the father killing the mother which resulted in a worse mood following the image. In the CG, one youth reported having an ID-image of a funeral of his/her grandmother, who had died after a long illness, without experiencing a mood change in reaction to this image. The remaining two adolescents of the CG reported images related to the death of close relatives connected with a negative altered mood, however, this indicates that the images reported by these two participants might not actually be ID-images according to the definition by Weßlau et al. [4]. Excluding these two adolescents, a re-analysis using the Fishers-Yates test showed that the difference between the 7 patients suffering from PTSD experiencing ID-images, versus the one participant experiencing ID-images from the control group, yielded a marginal statistical significance (*p* = .05). Due to the small sample size, however, we refrain from further quantitative analysis.

## Discussion

The aim of the current study was to gain comprehensive insights into the characteristics of positive MI, negative MI, as well as ID-images in an adolescent sample suffering from PTSD after childhood abuse. To our knowledge, this is the first study to assess the role of mental images in a clinical adolescent PTSD sample and to compare the results with a CG of adolescents without any mental disorder.

### Negative MI

In line with our hypotheses and what can be inferred from adult studies [7], negative mental images were present in more than 90% of the PTSD-patients, while only 25% of the adolescents without a mental disorder reported experiencing negative MI. Furthermore, as expected, the negative images were more vivid and more distressing, as well as being far more frequent, in traumatized adolescents compared to the CG adolescents with negative MI. Furthermore, in the PTSD group, the negative images were more strongly related to an autobiographical experience. These findings are also in line with studies on adolescents samples with other mental disorders such as social anxiety or depression [48, 56–58].

Unexpectedly, the level of controllability yielded no differences between the two groups, given that both described their negative images as rather uncontrollable. This indicates that the level of uncontrollability does not seem to be the differentiating characteristic regarding negative MI in adolescence. According to Burnett Heyes et al. [30], adolescents in general may not be cognitively capable of fully controlling MI, leading to a greater vulnerability regarding the distorting influences of negative MI. Thus, one may speculate that the ability to control intrusive imagery might reach a ceiling effect in adolescence, since even adolescents without a mental disorder are not yet sufficiently cognitively equipped to effectively control the images; this may possibly have led to there being no differences regarding the level of controllability between both groups. However, this hypothesis remains to be tested in future studies and, furthermore, our study may have also been underpowered in terms of detecting such a difference.

In line with our expectations, negative MI was found to be significantly related to greater feelings of disgust, helplessness, self-hate, anxiety, guilt, feelings of being contaminated or worthlessness in adolescents with PTSD, relative to adolescents without a mental disorder. Unexpectedly, no group differences emerged for the feelings of shame and sadness; this contradicts evidence from the literature on adult studies where it has been demonstrated that intrusive images correspond to hot spots of the trauma and are, thus, not only strongly related to emotions such as fear, guilt and anger, but also to shame and sadness (among others [11, 13, 59]). Nevertheless, our finding concurs with research on a non-clinical sample by Meiser-Stedman et al. [35], yielding significant relationships between the frequency of images and emotions such as sadness following a recent non-traumatic upsetting event in an adolescent community sample. Thus, one may speculate that shame and sadness could be common emotions in response to negative MI, even in adolescents without trauma history and a clinical diagnosis, which would then lead to a non-significant finding. On the other hand, we conducted statistical comparisons of 13 emotional states (described in the literature as potentially accompanying negative MI in PTSD) with Bonferroni-correction, which required a *p* value below .004 to reach significance. For the statistical analyses regarding *shame* and *sadness,* the *p* value was .01, indicating that a potential difference between the groups was lost due to the lack of adequate power and the simultaneous testing using Bonferroni-corrections of such a large number of emotions. There is an ongoing debate as to whether Bonferroni-correction is at all necessary and adequate in multiple testing (e.g., [60, 61]). Therefore, we refrain from discussing our results in greater depth regarding their content and recommend replicating these preliminary results in future studies before stating categorically that shame and sadness may not be relevant emotions in the realm of intrusive imagery in adolescent PTSD.

Participants were being asked for general changes in their mood following a negative image; the PTSD group reported their mood to be worse following negative MI whereas the CG reported a rather constant mood level. Against our hypothesis, this finding lacks statistical significance, indicating no differences between the two groups. This is surprising, since, as previously discussed, the PTSD group reported negative MI to be significantly more distressing as well as being connected with greater concomitant emotional states such as anxiety or helplessness. As this suggests that adolescents with PTSD present with stronger negative emotional responses to negative MI relative to healthy controls, we assume that our study might not have sufficient power to detect potential differences in mood alterations. In smaller samples as in the present study, it may also be useful to consider the effect size as this it often provides more reliable information because it is not biased by the sample size [62]. In this case, the effect size with *r* = −.23 indicates that the mood in the PTSD group was slightly worse compared to the CG following the negative image. However, since this was a rather small effect and, to the best of our knowledge, no comparable studies with a CG have been conducted, it remains unclear if a mood worsening following negative MI is a distinct characteristic in adolescent PTSD.

When examining the negative imagery perspective, we found that in both groups the images were rather experienced from a field-perspective (57.1% PTSD, 87.5% CG) than an observer-perspective (25% PTSD, 12.5% CG). 17.9% of the PTSD patients reported experiencing negative MI from both perspectives. This is an interesting result since research suggests that the perspective influences the emotional impact of the MI [63]. The numbers also correspond to those reported in PTSD studies in adults [7]. As reviewed in the introduction section, adult studies yielded mixed results regarding the perspective in intrusive imagery in adult PTSD. The field-perspective was associated with a higher intensity of emotional, physiological and psychological states (for a review see [1, 7]). Adult PTSD studies suggest that patients might adopt the observer-perspective to “strategically reduce emotion” ([7] , p. 225). As indicated above, adolescents may be less capable of using cognitive strategies such as changing the imagery perspective to protect themselves against the emotional impact [30], resulting in more field-perspective images in general. However, it seems that in other mental disorders, adolescents tend to use the observer-perspective, for instance in adolescent depression or social anxiety disorder (e.g., [48, 56, 57, 64]). More studies are needed to fully understand the role of the adopted imagery perspective in adolescent PTSD, while research is beginning to emerge with respect to the imagery perspective in non-clinical adolescent samples [63]. The latter indicates, for instance, that positive imagery training is more effective in cases of field-rather than observer-perspective imagery generation.

One may argue that the results regarding the differences in the presence and characteristics of negative images between the two groups constitute circular reasoning, since the presence of intrusive imagery is a core feature of PTSD diagnosis. However, re-experiencing symptoms do not necessarily have to consist of intrusive imagery in order to establish a PTSD diagnosis [32]. Thus, comparing negative MI in adolescent PTSD and non-traumatized adolescents seems to be an appropriate way to learn about differences between trauma-associated imagery in contrast to “usual” negative MI in adolescents.

### Positive MI

In contrast to our expectations, positive mental images were found in a comparable percentage of PTSD-patients (65.6%) and adolescents from the CG (71.9%). Moreover, the positive images were not found to be different with respect to the level of vividness, frequency, being linked to an autobiographical event or an alteration of mood in response to the images, yielding the amelioration of mood in both groups. Our findings regarding positive MI are in line with a study of 357 secondary school students [58], demonstrating that only depressive symptoms were related to less vivid, positive MI in adolescents; this association was not found for anxiety symptoms. Taken together, our results indicate that adolescent PTSD seems not to be related to an impoverished positive MI ability. However, since no other study has investigated the role of positive MI in traumatized adolescents, our findings are in need of replication. Studies in socially anxious and highly depressed adults suggest, for instance, that it might be an unbalanced ratio of positive-to-negative images and memories that are indicative of clinically relevant social anxiety or depression [4, 24]. In line with this, studies on depression have found that positive MI is biased in depressed adults and might be enhanced by a positive cognitive bias modification [8, 63]. The role of positive MI in PTSD is not well understood and needs broader empirical evidence before drawing definitive conclusions.

### ID-images

Our results suggest that ID-images are rather rare in adolescent patients suffering from PTSD and comparably frequent in adolescents without a mental disorder. However, after a more detailed qualitative analysis, it seems questionable whether the CG subjects correctly understood the concept of ID-images. There is tentative evidence that ID-images were more vivid in traumatized adolescents in our study, thus, the level of vividness might be the differentiating factor. However, in a large adult depression sample [4], the vividness of ID-images was not related to depression severity. Given the small sample size and the differing reported contents of ID images in the CG our results must be interpreted with caution and are in need of replication in larger samples.

### Limitations and further research

The results of the current study must be regarded as preliminary since they are constrained by several factors. Firstly, the sample sizes of the subgroups for the analyses of negative MI as well as ID-images were small and unequal, resulting in the use of non-parametric analysis or qualitative reports. Furthermore, the two groups differed in immigration background. Moreover, the PTSD group showed a significant lower educational level. Research in adult studies have shown similar tendencies with lower educational levels, as well as intelligence, in PTSD samples compared to non-PTSD samples [65]. Unfortunately, the use of non-parametric methods did not allow for more complex analyses controlling for the influence of educational level or immigration background. In addition, due to the lack of power associated with the small subsample sizes, we also had to refrain from the more complex analyses of interactions between, on the one hand, characteristics of MI and, on the one hand, PTSD symptoms.

Secondly, the retrospective nature of the study may have caused a social desirability response or memory bias, thus influencing our results [66, 67]. Ambulatory assessment strategies may, therefore, be useful for assessing MI and emotional as well as behavioral responses in adolescents when the imagery occurs. Thirdly, we cannot rule out that some participants may not have fully understood the concept of MI since it was only explained in a written description on the self-report measure. Clinical treatment practice shows that some adolescents require more explanation, at first, to understand what is meant by MI. Therefore, we cannot rule out that our results lack internal validity. However, the results of other studies examining mental images in child and adolescent samples [32, 35, 48, 68] which used self-report measures for the assessment of MI indicate that internal validity might not be threatened. Nevertheless, future studies should consider using additional semi-structured interviews to limit such influences on the results.

Lastly, the current PTSD sample consisted of victims of CA. Therefore, the generalization of the results regarding other trauma-types may be restricted and needs further research. Future studies should investigate a third group consisting of healthy non-PTSD adolescents with CA history in order to disentangle whether the differences found in our results depend on the clinical diagnosis of PTSD or on the potential abuse history.

Taken together, future research with larger sample sizes and a combination of self-report and semi-structured interviews should be used in order to assess further the role of negative MI, positive MI and ID-images in adolescents suffering from PTSD. Future studies should also consider the assessment of important peri-traumatic variables, such as dissociation, in order to extend our knowledge about the interaction of intrusive imagery and peri-traumatic factors as crucial components in the current models of the maintenance of PTSD in adults [12, 13, 59] as this may also be applicable to adolescent PTSD patients. Initial evidence in 10 to 12 year-old patients suffering from PTSD support the latter assumption [69]. Furthermore, the role of the imagery perspective in traumatized adolescents should be addressed since it appears that this is a crucial aspect regarding the effects of MI (e.g., the observer-perspective as an emotion regulation technique to dampen negative emotionality following intrusive imagery).

## Conclusion

At least for the experience of negative MI, our study provides evidence that this is an important feature of adolescent PTSD. Thus, interventions tailored to the developmental needs of adolescents and which target the adolescents’ intrusions by Imagery Rescripting techniques should be developed and examined. In adults, recent RCTs have found astonishing effect sizes for the use of Imagery Rescripting as a stand-alone treatment for abuse-related PTSD [70]. Moreover, CBM procedures for the augmentation of standard PTSD treatments for adult patients are about to emerge [71]. Initial evidence shows that positive CBM appraisal training, compared to negative CBM appraisal training, decreases distress associated with intrusions in adult participants who have experienced a stressful life event [72]. Furthermore, research suggests that positive imagery training may also be effective when considered as an adjunct to a standard cognitive behavioral treatment for PTSD in adolescents [63]. In a recent review, Schwarz et al. [73] found that interventions targeting MI can be effective in reducing the symptom severity in adolescent patients, however, there is still a significant need for more studies on the effectiveness of those interventions adapted in a youth-manner.

To summarize, the empirical evidence regarding the role of positive and negative MI, as well as ID-images, in adolescent patients suffering from PTSD remains sparse. Our study provides preliminary evidence supporting the crucial role of negative MI in traumatized adolescents. For the development of specific developmentally-adapted imagery-focused interventions for the treatment of adolescent PTSD symptoms, we need more studies to replicate and to extend our results in order to understand fully the interrelations between the distinct characteristics of negative MI such as the image perspective. The roles of positive MI and ID-images are not well understood in children and adolescents and require further investigation.

## Supplementary Information

**Additional file 1.**

## Data Availability

The datasets and materials generated during and/or analyzed during the current study are available from the corresponding author on reasonable request.

## Abbreviations

CA: Childhood sexual abuse and/or childhood physical abuse
CAPS-CA: Clinician Administered PTSD Scale – Child and Adolescent version
CBM: Cognitive bias modification
CG: Control group
D-CPT: Developmentally adapted Cognitive Processing Therapy
DSM-IV-TR: Diagnostic and Statistical Manual of Mental Disorders (4th edition, text revision)
GCTR: German Clinical Trial Registry
IBS-KJ: Interview zur Posttraumatischen Belastungsstörung bei Kindern und Jugendlichen [German CAPS-CA]
ID-images: Images of injury and death that lead to positive emotions
Kinder-DIPS: Diagnostic Interview for Mental Disorders in Childhood and Adolescence
M: Mean
MANOVA: Multivariate analysis of variance
Mdn: Median
MI: Mental imagery
PTSD: Posttraumatic stress disorder
RCT: Randomized controlled trial
SCID I: Structured Clinical Interview for DSM-IV Axis I
SCID II: Structured Clinical Interview for DSM-IV Axis II
SD: Standard deviation
UCLA-PTSD-RI: University of California Los Angeles PTSD Reaction Index for DSM-IV (Revision 1)
WL/TA: Wait-list condition with treatment advice
