# Supplementary material for: Mental imagery in adolescent PTSD patients after child abuse: a comparison with matched healthy controls
Source: BMC Psychiatry. 2022 Jan 27;22:64. doi: 10.1186/s12888-022-03706-8 (PMC8793273; doi:10.1186/s12888-022-03706-8)
Supplement: Supplementary file 1 — Additional file 1. [file 12888_2022_3706_MOESM1_ESM.pdf]

Additional File 1. Exploratory analyses between MI characteristics and psychopathologies in the PTSD group.

Supplementary Table 1. Spearman correlations (Spearman's ρ) between the number of comorbidities in the PTSD group and characteristics of negative MI.

	Frequency	Vividness	Mood alteration	Distress	Controllability	Autobiographical association
No of comorbidities	.346	.187	-.102	.241	-.031	-.064

Note: * p <.05, **significant after Bonferroni-correction: adjusted p = .05/6 = .008, n ranging from 29-27; No of comorbidities = number of comorbidities according to DSM-IV, Mood alteration = mood alteration in response to the negative image, distress= distress caused by the negative image.

Supplementary Table 2. Spearman correlations (Spearman's ρ) between the number of comorbidities in the PTSD group and characteristics of positive MI.

	Frequency	Vividness	Mood alteration	Autobiographical association
No of comorbidities	.054	.242	-.281	.008

Note: * p <.05, **significant after Bonferroni-correction: adjusted p = .05/4 = .013; n = 21; No of comorbidities = number of comorbidities according to DSM-IV, Mood alteration = mood alteration in response to the positive image.

Supplementary Table 3. Spearman correlations (Spearman's ρ) between the number of comorbidities in the PTSD group and characteristics of ID images.

	Frequency	Vividness	Mood alteration
No of comorbidities	-.889*	.121	-.606

Note: * p <.05, **significant after Bonferroni-correction: adjusted p = .05/3 = .017; n ranged from 7-6; No of comorbidities = number of comorbidities according to DSM-IV, Mood alteration = mood alteration in response to the ID image.

Supplementary Table 4. Spearman correlations (Spearman's ρ) between abuse characteristics in the PTSD group and characteristics of negative MI.

	Frequency	Vividness	Mood alteration	Distress	Controllability	Autobiographical association
**Physical abuse**						
No of clusters	.877	.270	-.243	.049	-.254	-.051
Age at onset	.246	.154	-.054	.083	-.245	.377
Duration	-.080	.023	-.013	.037	.073	-.338
**Sexual abuse**						
No of cluster	.137	.396*	-.253	.228	-.244	.084
Age at onset	-.043	.280	.019	.158	.119	.231
Duration	.201	.078	-.287	.076	-.226	-.354

Note: * p <.05, **significant after Bonferroni-correction: adjusted $p = \frac{.05}{6*6} = .001$; n ranging from 18-28; Mood alteration = mood alteration in response to the negative image, distress= distress caused by the negative image; duration = duration in months.

Supplementary Table 5. Spearman correlations (Spearman's ρ) between abuse characteristics in the PTSD and characteristics of positive MI.

	Frequency	Vividness	Mood alteration	Autobiographical association
**Physical abuse**				
No of clusters	-.216	-.447	-.156	-.249
Age at onset	.084	-.340	-.151	.010
Duration	-.121	.357	.100	.110
**Sexual abuse**				
No of clusters	-.118	.027	-.226	-.110
Age at onset	.187	-.086	.021	.056
Duration	.080	.128	-.111	.075

Note: * p <.05, **significant after Bonferroni-correction: adjusted $p = \frac{.05}{4*6} = .002$, n ranging from 15-21; Mood alteration = mood alteration in response to the positive image, duration = duration in months.

Supplementary Table 6. Spearman correlations (Spearman's ρ) between abuse characteristics in the PTSD group and characteristics of ID images.

	Frequency	Vividness	Mood alteration
**Physical abuse**			
No of clusters	-.188	.493	**-.985****
Age at onset	.316	.800	-.316
Duration	-.316	-.800	.316
**Sexual abuse**			
No of clusters	-.575	.429	-.772
Age at onset	-.031	.544	-.411
Duration	-.216	.632	-.822*

Note: * p <.05, **significant after Bonferroni-correction: adjusted $p = \frac{.05}{3*6} = .003$; n ranged from 4-7; Mood alteration = mood alteration in response to the ID image, duration = duration in months.

Supplementary Table 7. Pearson's product-moment correlations between PTSD symptoms and characteristics of negative MI in the PTSD group.

	Frequency	Vivid-ness	Mood alteration	Distress	Control-lability	Autobiographical association
CAPS-CA sum	.502*	.162	-.358	.402*	-.273	-.341
Re-experiencing	.572**	.239	-.333	.390*	-.412*	-.101
Avoidance	.433*	.217	-.317	.367	-.344	-.295
Hyperarousal	.513*	.084	-.380*	.437*	-.136	-.349

* p <.05, **significant after Bonferroni-correction: adjusted $p = \frac{.05}{6*4} = .002$, n ranging from 27-29; Mood alteration = mood alteration in response to the negative image, distress= distress caused by the negative image; CAPS-CA = Clinician-Administered PTSD Scale for Children and Adolescents.

Supplementary Table 8. Pearson's product-moment correlations between PTSD symptoms and characteristics of positive MI in the PTSD group.

	Frequency	Vividness	Mood alteration	Autobiographical association
CAPS-CA sum	.084	.266	-.592*	.109
Re-experiencing	.219	-.119	-.169	-.003
Avoidance	.106	.294	-.615*	.014
Hyperarousal	-.052	.253	-.535*	.210

Note: * p <.05, **significant after Bonferroni-correction: adjusted $p = \frac{.05}{4*4} = .003$, n =21; Mood alteration = mood alteration in response to the positive image, CAPS-CA = Clinician-Administered PTSD Scale for Children and Adolescents.

Supplementary Table 9. Pearson's product-moment correlations between PTSD symptoms and characteristics of ID images in the PTSD group.

	Frequency	Vividness	Mood alteration
CAPS-CA sum	-.184	.423	-.887*
Re-experiencing	-.329	.414	-.779
Avoidance	-.325	.472	-.838*
Hyperarousal	.195	.223	-.758

Note:* p <.05, **significant after Bonferroni-correction: adjusted $p = \frac{.05}{3*4} = .004$, n ranged from 6-7; Mood alteration = mood alteration in response to the ID image, duration = duration in months. CAPS-CA = Clinician-Administered PTSD Scale for Children and Adolescents
